# Glutamine Repeat Variants in Human RUNX2 Associated with Decreased Femoral Neck BMD, Broadband Ultrasound Attenuation and Target Gene Transactivation

**DOI:** 10.1371/journal.pone.0042617

**Published:** 2012-08-13

**Authors:** Nigel A. Morrison, Alexandre A. Stephens, Motomi Osato, Patsie Polly, Timothy C. Tan, Namiko Yamashita, James D. Doecke, Julie Pasco, Nicolette Fozzard, Graeme Jones, Stuart H. Ralston, Philip N. Sambrook, Richard L. Prince, Geoff C. Nicholson

**Affiliations:** 1 School of Medical Sciences, Griffith University, Southport, Australia; 2 Institute of Molecular and Cell Biology, Singapore, Singapore; 3 Department of Pathology and Inflammation and Infection Research Centre, School of Medical Sciences, University of New South Wales, Sydney, Australia; 4 Cancer Science Institute of Singapore, National University of Singapore, Singapore, Singapore; 5 School of Medicine, Deakin University, Geelong, Australia; 6 The Menzies Institute, Hobart, Australia; 7 Centre for Molecular Medicine, The University of Edinburgh, Edinburgh, Scotland; 8 Royal North Shore Hospital, The University of Sydney, Sydney, Australia; 9 Western Australian Institute of Medical Research and the Department of Endocrinology and Diabetes, Sir Charles Gairdner Hospital, Perth, Australia; 10 Rural Clinical School, School of Medicine, The University of Queensland, Toowoomba, Australia; University of Liverpool, United Kingdom

## Abstract

RUNX2 is an essential transcription factor required for skeletal development and cartilage formation. Haploinsufficiency of RUNX2 leads to cleidocranial displaysia (CCD) a skeletal disorder characterised by gross dysgenesis of bones particularly those derived from intramembranous bone formation. A notable feature of the RUNX2 protein is the polyglutamine and polyalanine (23Q/17A) domain coded by a repeat sequence. Since none of the known mutations causing CCD characterised to date map in the glutamine repeat region, we hypothesised that Q-repeat mutations may be related to a more subtle bone phenotype. We screened subjects derived from four normal populations for Q-repeat variants. A total of 22 subjects were identified who were heterozygous for a wild type allele and a Q-repeat variant allele: (15Q, 16Q, 18Q and 30Q). Although not every subject had data for all measures, Q-repeat variants had a significant deficit in BMD with an average decrease of 0.7SD measured over 12 BMD-related parameters (p = 0.005). Femoral neck BMD was measured in all subjects (−0.6SD, p = 0.0007). The transactivation function of RUNX2 was determined for 16Q and 30Q alleles using a reporter gene assay. 16Q and 30Q alleles displayed significantly lower transactivation function compared to wild type (23Q). Our analysis has identified novel Q-repeat mutations that occur at a collective frequency of about 0.4%. These mutations significantly alter BMD and display impaired transactivation function, introducing a new class of functionally relevant RUNX2 mutants.

## Introduction

Osteoporosis is the major metabolic bone disease among developed nations. The disease is characterized by low bone density and reduced bone quality through the deterioration of bone micro architecture. As a consequence, sufferers have impaired skeletal strength and an increased susceptibility to osteoporotic fractures. Bone mineral density (BMD) is a complex trait and is controlled by environmental and genetic factors [1]. BMD is a primary predictor of osteoporotic fractures; it is however a continuous trait related to age and weight. Osteoporosis as a category is a truncation of this continuous normally distributed trait, defined by a BMD value less than 2.5 SD from the population mean of young adults [2]. The proportion of fractures attributable to osteoporosis ranges from 10%–44% [3].

RUNX2 is a key regulator of skeletogenesis, bone and cartilage formation [4]–[8] and has been genetically associated with BMD [9]–[15]. RUNX2 transactivates genes such as osteocalcin, type 1 collagen and osteopontin and is initially expressed in mesenchymal condensations where it plays an essential role in osteoblast differentiation and in chondrocyte maturation [16]. RUNX2 deficient mice have un-mineralized skeletons with few immature osteoblasts and an absence of vascular and mesenchymal cell invasion in the cartilaginous templates. Heterozygous mutations in coding and promoter sequences of RUNX2 cause the dominantly inherited skeletal syndrome cleidocranial dysplasia (CCD). The disorder is characterised by persistently open or delayed closure of sutures, hypoplasia/aplasia of clavicles, Wormian bones, supernumerary teeth, short stature and other skeletal abnormalities [17], [18]. A spectrum of severity of CCD symptoms is correlated with the extent of transcriptional activity remaining in the cognate mutant RUNX2 with severest symptoms associated with complete loss of RUNX2 function [19] and milder symptoms correlated with some residual RUNX2 transactivation function [20].

RUNX2 has consecutive polyglutamine and polyalanine repeats (Q/A repeat) in the protein sequence. Such repeat regions have the capacity to mutate via strand slippage during DNA replication. Glutamine repeat sequence expansion has been the cause of some diseases that show genetic anticipation, where severity increases in subsequent generations as repeat length increases due to errors in replication [21]. Wild type human RUNX2 contains a 23Q/17A repeat; 23 consecutive glutamine followed by 17 alanine residues. An insertion of the polyalanine tract (23Q/27A) was observed in one patient with CCD [7] although no further polyalanine-related CCD patients have been reported and no evidence currently associates the Q-repeat region with CCD. We previously identified Q-variants (15Q, 16Q, 24Q and 30Q) in an Australian fracture cohort [9] and two 16Q variants as well as a single alanine expansion variant (23Q/23A) in a randomly selected population from Aberdeen [10]. Pineda et al. [22] observed three Q-variants (16Q, 18Q and 30Q) in a Spanish population. Evidence exists that the RUNX2 Q-repeat is a site of functional variation; in carnivores the length of the Q repeat is significantly associated with mid-face length and nose curvature [23], indicating an effect on bone growth rates. Sears et al. [24] confirmed the functional nature of carnivore related Q/A repeat alterations, showing correlation of transcriptional variation with facial length in carnivores. These lines of evidence suggest that RUNX2 repeat variation may be functionally different in the human population. We hypothesised that glutamine repeat variants would exist in normal populations and may influence adult BMD and/or risk of fracture.

## Materials and Methods

### Study Subjects

The study subjects were obtained from four different epidemiological studies of bone density and are summarised in Table 1. All subjects were female. Voting in elections in Australia is compulsory and each region maintains an electoral roll. The West Australian study [25] consisted of females between the ages of 70 and 85 years who were randomly ascertained from the electoral role and approached by letter. The Geelong Osteoporosis study comprised a cohort recruited at random from the electoral role (GOS random) [26] and a specific fracture study (GOS fracture), where all fracture cases over the age of 35 years presenting at the only two radiology practices in the region were invited to join, as described [27]. Genotype was obtained on 834 subjects with no history of fracture from the random sample of GOS and 578 of the GOS fracture cases. The Sydney study comprised monozygotic and dizygotic twins who volunteered for studies of general medical issues through the Australian twin registry, as described [28]. Self reported history of fracture was available among a series of questionnaires completed by volunteers. Data were obtained on 980 participants. In the Tasmanian older adult cohort (TASOAC) study, 388 DNA of females were available of which 385 yielded genotype data. In this study, subjects ranged from 50 to 80 and were recruited by random ascertainment from the electoral roll [29]. For the Aberdeen study, subjects were postmenopausal women aged between 45 and 55 years who were approached at random using Community Health Index records of Aberdeen, Scotland as previously described [30]. Data were obtained on 991 study participants. In addition, 101 elderly female clinic patients with established osteoporosis and at least one osteoporotic fracture were available from the Geelong endocrine clinic. A similar cohort of 200 clinical patients with established osteoporosis defined by recurrent vertebral fracture was available from a New Zealand study, as described [31], [32]. BMD data were not available for clinic patients. Individuals in all studies were female Caucasians and no subjects were excluded. Appropriate written consent was obtained from subjects under procedures in accordance with the Declaration of Helsinki approved by the relevant human research ethics committees (HREC) as previously described [25]–[32]. The committees are as follows: Sir Charles Gairdner Hospital HREC, Barwon Health HREC, Northern Sydney Local Health District HREC, Southern Tasmanian Health and Medical HREC and University of Otago HREC.

**Table 1:** Characteristics of population studies.

Study	Age range	N genotyped	Recruitment via	Reference
Western Australian	70–82	1078	Electoral roll	25
GOS random	20–92	822	Electoral roll	26
GOS fracture	35–95	598	Present with fracture	27
Sydney	19–78	980	Twin registry	28
TASOAC	50–80	385	Electoral roll	29
Aberdeen	45–55	991	Health records	30

### Bone measures

Bone density was measured by dual energy X-ray absorptiometry (DEXA). In the Aberdeen study a Norland XR26 or XR36 was used (Norland Corporation, Fort Atkinson, WI, USA). A Lunar DPX-L machine was used for the GOS. The Western Australian study used a Hologic 4500A fan beam densitometer to measure total BMD of the hip region. A Lunar Achilles ultrasound machine was used to measure the left calcaneus bone. Measures of speed of sound (SOS), broadband ultrasound attenuation (BUA) and bone stiffness were available. In the Sydney study, a Hologic 4500A fan beam densitometer was used to measure BMD at the lumbar spine, total hip, femoral neck and whole body. Western Australian, TASOAC and Sydney studies used the same type of densitometer and were calibrated with the same standard phantoms.

### Detection of Q-variants

PCR was used to amplify RUNX2 exon 1 fragments harboring the Q/A repeat using the primers forward 5′-CCGGCAAAATGAGCGACG-3′ and reverse 5′-GGGCGGTGTAGCCTCTTACCTT-3′. The PCRs were carried out in 20 µl reactions containing 5×10−7 M primers, PCR reaction buffer, 125 µM dNTPs, 80 ng genomic DNA, 0.5 units *Taq* polymerase in 20 µl as specified by the supplier (Promega, Sydney, Australia). The study populations were genotyped separately by differing means: testing 100 random DNA by all methods gave the same genotypes. For the GOS and TASOAC, the resulting 336 bp fragments were resolved via nondenaturing 10% polyacrylamide gel electrophoresis (PAGE). Heteroduplex analysis was used to determine the presence of Q-repeat variants. For the Aberdeen populations the PCR fragment was digested with MspA1I (New England Biolabs) and resolved on 3% agarose. For the Western Australian samples, all PCR products were initially analysed via dHPLC (Varian Prostar 430, Varian Industries, Sydney, Australia) and subsequently by heteroduplex PAGE. Genotyping was done twice for all samples and all variant genotypes confirmed by PAGE to reveal heteroduplexes. All PCR amplified DNA fragments that displayed unique mobility patterns on PAGE were sequenced using BigDye Terminator v1.1 ready mix according to the manufacturer's protocol (Applied Biosystems).

### Plasmids, transfection and cell culture

Expression plasmids containing mutant Q/A domains were constructed by cloning the *XbaI-XbaI* fragment from full length RUNX2 expression plasmid pEF-αA [33] into pUC18 to create pUC18RUNX2 which served as a template to create glutamine variants via PCR cloning. PCR was used to amplify partial RUNX2 promoter and exon 1 fragments from DNA samples harboring Q/A mutations using the oligonucleotide primers 5′-TTCACCACCGGACTCCAACT-3′ for the 5′ side and 5′-CATCTGGTACCTCTCCGAGGGCTACCACCTTGAAGGCCACGGGCAGGGTC-3′ for the 3′ side. The reverse primer contained an *EcoNI* tag facilitating the cloning of the PCR amplified product into the *BglII-EcoNI* site of pUC18RUNX2. Mutant Q/A regions were confirmed by DNA sequencing and the *XbaI-XbaI* fragments were cloned into the *XbaI-XbaI* site of the mammalian expression vector pEF-BOS [34]. Restriction digest analysis and DNA sequencing confirmed the orientations of RUNX2 inserts. pGL3-Basic served as a template to create the reporter constructs. *BglII* and *HindIII* sites were introduced by PCR amplification in the human osteocalcin gene using oligonucleotide primers 5′-CAGGAGATCTCTGACCGTCGAGCTG-3′ for the 5′ side and 5′-GGGCAAGCTTGGTGTCTCGGGTGGC-3′ for the 3′ side. The resulting *BglII-HindIII* fragment was cloned into the corresponding sites of pGL3-Basic to create pOSLUC, which has 590 basepairs of the human osteocalcin promoter, including 70 base pairs of the untranslated message. Oligonucleotides containing a consensus mouse osteocalcin RUNX2 response element (RRE: 5′-GATCCGCTGCAATCACCAACCACAGCA, with RUNX2 consensus binding site underlined) were cloned into the *BglII* site of pOSLUC to create pRRE, which had three copies of the RRE inserted as direct repeats. Transfections were performed using FuGENE (Roche) as the manufacturer's protocols. Antibody immuno-staining was performed as described in Yoshida et al. [35]. 1,25 dihydroxyvitamin D_3_ (1,25(OH)_2_D_3_) was kept in the dark under argon in isopropanol and added with appropriate vehicle controls after dilution in ethanol. No more than 0.1 µl ethanol per ml medium was present in cell culture. Western blots of transfected cells were done by standard methods using anti-RUNX2 antibody (D130-3, MBL International) according to the manufacturer's instructions.

### Luciferase Assay

NIH3T3 cells and HEK293 cells were maintained in DMEM (Gibco) supplemented with 10% FBS (v/v) (Gibco), 1% Penicillin-Streptomycin (v/v) (Gibco) in a 5% CO_2_ humidified atmosphere at 37°C. For the luciferase assay NIH3T3 cells were seeded into 6-well plates at a density of 1×10^5^ cells/well 24 hours prior to transfection. HEK293 cells were seeded into 12-well plates at a density of 1×10^5^ cells/well 24 hours prior to transfection. NIH3T3 and HEK293 cells were transfected using FuGENE6 transfection reagent according to the manufacturer's instructions (Promega). Cells were harvested 48 hours post-transfection. Luciferase activities were determined using the dual luciferase assay system described by Dyer et al. 2000 [36]. pRL-CMV (Promega) was used as an internal control to normalize results.

### Gel shift assays, *in vitro* protein translation and GST pull-down assays

Gel shift assays were done by standard methods as previously described, using 5% PAGE and *in vitro* translated proteins [37]. DNA probes representing RUNX2 binding sites were end labelled using a fill in reaction of 5′ overhangs using DNA polymerase 1 Klenow fragment and fragments purified by PAGE. *In vitro* translated human RUNX2 variant proteins 23Q wild type, 16Q-variant, and 30Q-variant were generated from DNA fragments representing 23Q, 16Q, or 30Q variants recloned into a T7 promoter containing hRUNX2 vector by using T7 RNA polymerase (TNT® Promega, USA) and coupled transcription-translation reactions containing [^35^S]-methionine. Protein products were visualised and quantified by [^35^S]-methionine incorporation. Bacterial over-expression of glutathione-S-transferase coupled human VDR (GSThVDR) fusion protein was performed by induction with isopropyl-β-D-thio-galactopyranoside (IPTG, 1.25 mM) for 3 h at 30°C in the JM109 *E.coli* strain as described previously [37]. GST pull-down assays were performed by incubation of GSThVDR with either [^35^S]-23Q RUNX2 wild type, [^35^S]-16Q variant, [^35^S]-30Q variant RUNX2. As a binding agent, a GSThVDR-Sepharose bead slurry (50% w/v of beads) in PPI buffer (20 mM HEPES, pH 7.9; 200 mM KCl; 1 mM EDTA, 4 mM MgCl2, 1 mM dithiothreitol, 0.1% Nonidet P-40 and 10% glycerol) was used. GST fusion-Sepharose slurries were pre-blocked in PPI buffer containing bovine serum albumin (1 µg/µl) prior to use in pull-down assays. Unbound [^35^S]-23Q RUNX2 wild type, [^35^S]-16Q variant or [^35^S]-30Q variant RUNX2 labelled proteins were washed away with PPI buffer. *In vitro* translated [^35^S] 23Q, [^35^S] 16Q or [^35^S] 30Q that had bound to GSThVDR-Sepharose was released from the Sepharose, electrophoresed through a 12% SDS-PAGE, and detected by autoradiography using either phosphor screens (BioRad) or X-ray film. Densitometric analysis on resolved bands was performed using the ChemicDoc system (BioRad).

### Statistical methods

Within studies, Q-variants were analysed using analysis of variance (ANOVA) and Student's T-test. Genotype status (Q-variant carrier or not) was coded as one and zero, respectively, and used as a variable in ANCOVA analysis to test the effect of covariates such as age and weight. Age, age-weight and age-weight^−1^ adjusted values of bone density parameters were produced using linear regression. Stiffness was log_e_ transformed to comply with equality of variance assumptions in ANOVA. These adjustments had no material influence on the conclusions and Z scores of age-weight^−1^ regression are presented. Incident fracture during five years of observation was available for the Western Australian study. Incident fracture was categorised as zero and one for absence and presence of fracture, respectively, for analysis by logistic regression and the number of such fracture events also examined in genotype groups using contingency tables. Results of logistic regression are presented as p values and odds ratios (OR) with 95% confidence intervals (CI). Genotypes were examined pooled (all variants) and separately as either deletions or insertions. The only measure in common across all studies was femoral neck BMD. In order to pool all studies, each individual was expressed within a study as a Z-score of residual femoral neck BMD from the age-weight^−1^ regression specific for that study. Under the null hypothesis of no effect at this locus on BMD, the Z scores should be distributed randomly around a mean of zero. Population simulations were constructed in excel, using the Monte Carlo simulation plugin, PopTools [38], and the correlated variable tool to simulate bone density parameters as a series of correlated normal distributions, based on the correlations observed in the populations studied. Results of transfections were analysed using ANOVA with Fisher's least significant difference test for pair wise comparisons. Counts of cells were analysed using contingency tables and Chi-square. Analysis of covariance was used to analyse categorical and continuous covariates.

## Results

### Q-variants identified

From the 1078 individuals genotyped in the Western Australian study, there were five 16Q alleles, one 18Q allele and two 30Q alleles. In the GOS random population sample three Q-variants were identified in 822 subjects: these were one 15Q and two 16Q variants. In the GOS fracture study, where recruitment was based on any fracture at any age, four Q-variants were identified in 598 subjects: these were two 16Q and two 30Q variants. In a prior publication [9], three of these variants were reported (two 16Q and one 30Q): in this study more samples from the GOS fracture cohort were genotyped and an additional variant identified. In the Sydney population of 980, five individuals with Q-variants were identified: carrying 16Q, 18Q and 30Q. The 16Q and 18Q variants were found in two pairs of monozygotic twins. The 30Q variant was in one individual of a dizygotic twin pair. For analysis, the average bone density values of the monozygotic twin pairs were taken and the pair considered as a single genetically unique individual. In the Tasmanian study (TASOAC) no Q-variants were found in 385 genotyped. In the Aberdeen study, two 16Q variants were identified previously from 991 subjects [10]; in this study their bone parameters are reported. In order to estimate the frequency of the Q-variants without bias, only those populations where the recruitment strategy involved volunteers were considered (Western Australia, Geelong, Tasmania and Aberdeen). In this case, 13 Q-variants were observed within 3276 subjects, yielding a population frequency estimate of 0.004 (95%CI, 0.002 to 0.007). Using the binomial theorem and the observed frequency of Q-variants, it is estimated that 95% of repeat studies of a similar size (approximately 3000 subjects) will detect between 6 and 19 such Q-variant carriers, if allele frequency is similar in other populations. In 103 female post-menopausal clinic patients with established osteoporosis with fracture, 16Q and 30Q variants were found. In a similar population of vertebral fracture cases from New Zealand [31], [32], two 30Q variants were found in 200 patients genotyped. Therefore, in clinic patients with osteoporosis, four Q-variants were found in 303 patients genotyped. Apart from those Q-variants described above, three examples of 24Q alleles were detected overall. These 24Q subjects were not considered in this analysis. All Q-repeat variants were heterozygous carriers with the other chromosome containing a 23Q/17A wild type allele.

### Bone density in Q-variants

**Figure 1:**
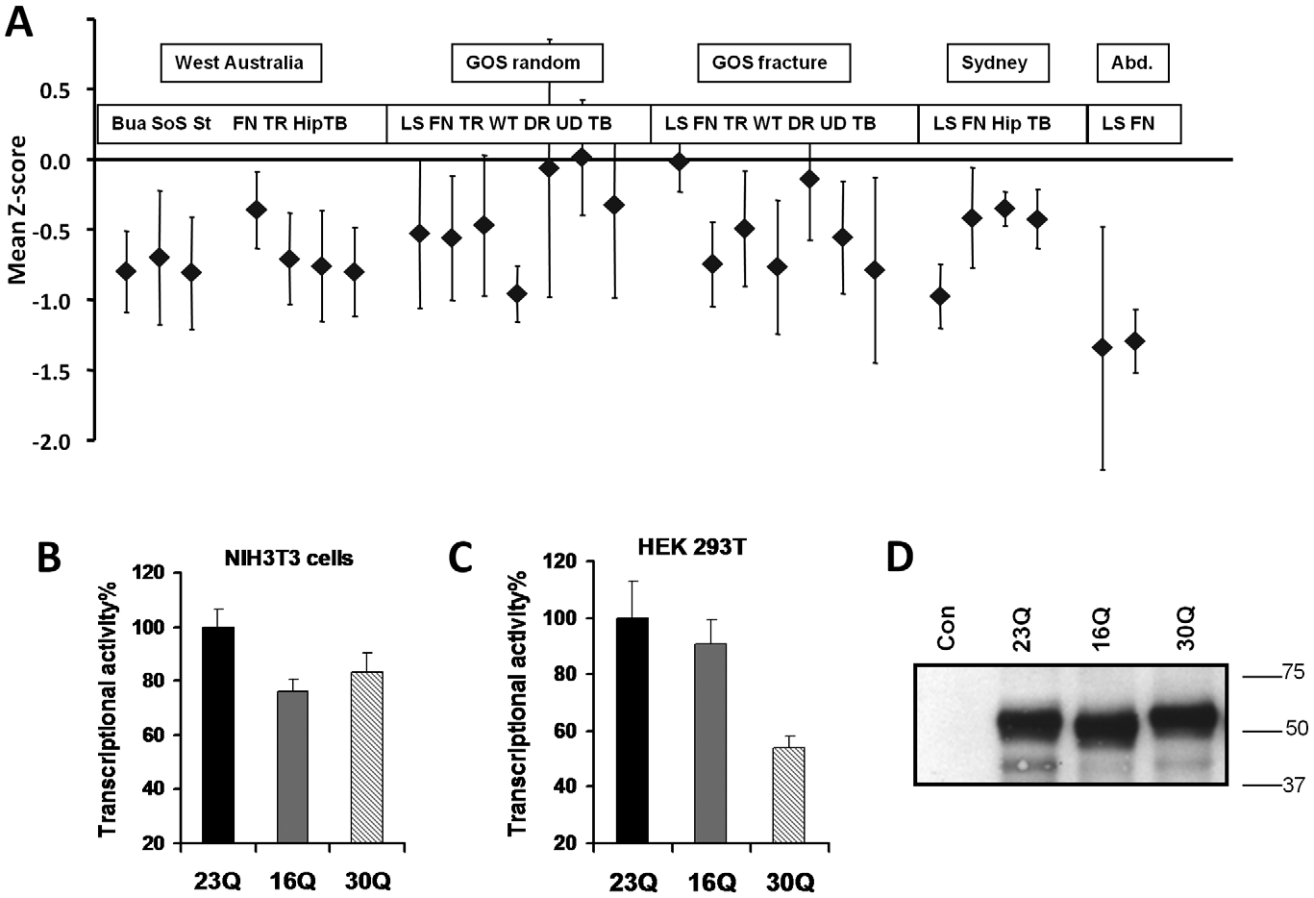
Characterization of RUNX2 Q variants with respect to clinical measures of bone density and *in vitro* transactivation. **A.** RUNX2 Q-variant carriers from Western Australia, Geelong (GOS random sample and GOS fracture), Sydney and Aberdeen studies have lower mean bone density parameters. Q-variants have lower mean Z-scores of bone density measured by calcaneal ultrasound (BUA, SOS, Stiff) and by DEXA at various skeletal sites. Abbreviations: Abd., Aberdeen; BUA, broadband ultrasound attenuation; SOS, speed of sound; Stiff, bone stiffness; FN, femoral neck BMD; TR, trochanteric region BMD; Hip, Hip BMD; TB, total body BMD; LS, lumbar spine BMD; WT, Ward's triangle BMD; DR, distal radius BMD; UD, ultradistal radius BMD. Error bars are standard error. Z-scores presented are the within-study age-weight^−1^ adjusted Z score derived from within that cohort. **B** and **C**. Q-variant expression vectors have diminished capacity to transactivate target promoters in NIH3T3 mouse fibroblasts (**B**) and human embryonic kidney cell (HEK293) (**C**). Cells were transfected as described in the methods. In **B** the target was pRRE which is the human osteocalcin promoter with three additional synthetic mouse RUNX2 target sites added upstream, and in **C** the target promoter construct was 590 bp of the authentic human osteocalcin promoter driving luciferase (pOSLUC). **D.** Transfected cells show appropriate sizes of expressed product and exhibit equivalent amount of protein. NIH3T3 cells were transfected with empty vector (Con) or expression constructs as indicated. Cellular extracts were Western blotted after electrophoresis on SDS-PAGE. Numbers indicate molecular weight markers in kilodaltons. In **B** and **C** transactivation is expressed as percentage relative to the 23Q variant in order to normalise scales of NIH3T3 and HEK293 cells.

Characteristics of Q-variant carriers with respect to femoral neck (FN) Z scores are presented in Table 2. Figure 1 shows the mean Z score of Q-variants for each measured parameter in each study cohort. The Q-variant carriers in Western Australian were compared to non carriers (N = 1021) for differences in the ultrasound measures of bone density: broadband ultrasound attenuation (BUA) and speed of sound (SOS). Q-variants had significantly lower BUA measures (n = 8, BUA, p = 0.025) at the calcaneus, with a difference of −0.80 standard deviations (SD, or Z score) and significantly lower SOS (p = 0.05) and log stiffness (p = 0.013). Weight, height and age were not significantly different in Q-variants. For all BMD values, age and age-weight^−1^ adjustment was done using regression and adjusted values produced. Individual values were then expressed as Z scores around the relevant population mean. While the particulars of reported measures change depending on such adjustment, the general outcomes reported here are not dependent on this mathematical adjustment. Measures of bone density using DEXA were similarly lower in Q-variants (Fig. 1A) although only nominally significant with the total hip BMD measure (p = 0.028). At other sites there was a trend for Q-variants to have lower BMD compared with all others in the study: femoral neck (p = 0.22), trochanter (p = 0.065), and intertrochanteric region (p = 0.06). Of the seven Q-variants identified from GOS there was a trend for negative Z scores (BMD below the population mean value, Fig. 1A) with 6 of 7 sites having negative average BMD. The Q-variant subjects from the Aberdeen study had negative Z-scores for both femoral neck and lumbar spine BMD (averaging −1.39SD and −1.36SD, p = 0.01 and p = 0.11 respectively, Fig. 1A). In the Sydney study, the data from each monozygotic twin pair were averaged and thus three were available with BMD data. The Q-variants had negative BMD Z-scores for the femoral neck with an average effect of −0.62SD. There was a trend for negative Z scores for all bone measures (Table 3).

**Table 2:** Characteristics of Q-variant allele carriers.

Allele	Age	Weight	Height	Z-FN
15Q	41	69	159	−0.33
16Q	30	79	171	−1.42
16Q	45	56	166	0.07
16Q	47	68	174	−1.52
16Q	49	66	161	−1.07
16Q*	69	71	165	−0.15
16Q	70	56	152	−0.34
16Q	72	80	168	0.89
16Q	75	71	165	−1.69
16Q	76	52	156	−0.91
16Q	78	61	152	−0.70
16Q	79	65	167	−0.31
16Q	85	70	147	−0.13
18Q*	65	66	161	−0.21
18Q	74	69	156	−0.24
30Q	39	97	161	−1.34
30Q	48	62	160	−1.37
30Q	72	57	161	0.07
30Q	74	54	162	−0.02
30Q	87	50	155	−1.18

Age (years), weight (kg), height (cm) and Z-score of femoral neck BMD are presented. Subjects are ranked by allele length and then age.

* indicates average of identical twin pair. In addition, a 16Q and three 30Q variants were observed in clinic patients.

**Table 3:** Bone parameters in standardized Z scores averaged over all Q-variants carriers.

Site	Mean Z	N	P value
Z_Spine	−0.709	14*	0.0032
Z_FN	−0.601	22*	0.0006
Z_WT	−0.484	6	0.1449
Z_TR	−0.774	14*	0.0022
Z_UD	−0.102	5	0.8358
Z_MI	−0.271	5	0.4149
Z_TB	−0.531	10*	0.0844
Z_INT	−0.760	7	0.0206
Z_HIP	−0.653	11*	0.0146
BUA	−0.798	8	0.0254
SOS	−0.700	8	0.0505
STIFF	−0.809	8	0.0130

N is the number of observations available.

* Asterisk indicates sites with data from more than one study combined using Z scores. P value is the test of the hypothesis that the mean Z score is zero. Key: Z_ indicates a Z score adjusted by age weight^−1^ regression, FN is femoral neck, WT is Ward's triangle, TR is trochanteric region, UD is ultra distal radius, MI is Mid shaft of the radius, TB is total body, INT is intertrochanteric region, HIP is total hip, BUA is broadband ultrasound attenuation measured at the calcaneus, SOS is speed of sound, STIFF is stiffness and p value refers to log_e_ transformed stiffness.

### Combining BMD data using Monte Carlo simulation

Bone density measures at different anatomical sites are correlated: additional measures in individuals therefore contribute useful information to explore the bone phenotype of Q-variants. All these data from skeletal sites can be used collectively to explore the genotype-phenotype relationship. Empirical p values based on Monte Carlo modelling make use of the additional data available in bone studies, where the scanning methods provide BMD measures of multiple sites. Considering only Z scores derived from within each study eliminates the effect of using different DEXA devices. A simulation was made based on the likelihood of sampling such individuals with measurements of correlated bone related variables. Correlated variables were simulated based on the measured correlations of bone parameters in the various populations, and a simulation of sampling was done in order to determine how many such samples met or exceeded the observed data. This approach was first taken with the Western Australian study, considering just deletion variants. There were six deletion variants, identified in a population sample of 1078 subjects, each with seven measured parameters related to bone. In this case, of 100,000 simulations of sampling six individuals measured for seven correlated bone parameters, only 252 such samples had an overall group mean equal to or more negative than the observed mean of −0.74 Z scores for all variables. This yields an empirical multivariate p value of 0.0025. The GOS study came from a similar population via similar recruitment with seven bone related parameters measured; although the bone parameters were measured using different equipment. The three deletions observed in GOS study were combined with those from Western Australia and a simulation done based on the idea of sampling from the combined population: in this case the observed mean was −0.63 Z scores and 235 samples from 100,000 trials met or exceeded the observed mean, giving an empirical p value of 0.0024. When 30Q variants were combined with all other variants from the two Australian studies the Monte Carlo based multivariate p value was: 0.0007. Of the 75 bone related measures from the Q variants identified in Aberdeen, Western Australia and Geelong studies, 59 measures had negative Z score values. Combining data from the three studies for Monte Carlo simulation resulted in 9 samples in 100,000 trials; giving an empirical probability of p = 0.0009. These simulations suggest that the additional measures provide useful data supporting the hypothesis that Q-variant carriers have lower bone density and that the average deficit is of the order of 0.7 Z scores of age, weight^−1^ adjusted BMD.

Using BMD data from the Q-variants derived from the GOS fracture cohort is not necessary, since a significant p value was already obtained using data from studies with no apparent recruitment bias. However, if the GOS fracture and Sydney cohort data were used in the Monte Carlo simulations the result was still significant. Addition of all subjects for whom BMD data were available to the Monte Carlo simulation supported the hypothesis that Q-variant carriers have significantly lower BMD (3 samples in one million trials with mean values equal to or more extreme than the observed means).

Femoral neck BMD was the only measure in common between the GOS random sample, GOS fracture, Aberdeen, Sydney and Western Australian studies. In order to combine the effects of these studies, age-weight^−1^ adjusted Z-scores for each study were taken giving a mean Z score of −0.60. Simply combining all populations, as if there were one global population from which the samples were drawn, gave a p value of 0.0007. The conclusions were not altered appreciably by taking the average of those who were twins.

### Relationship to fracture

In the GOS study, the random sample that was genotyped excluded any with history of fracture, whereas in the Western Australian study, although recruitment was essentially random, fracture was not a reason for exclusion. Of the eight Q-variants found in the Western Australian study, four had prior osteoporosis related fractures (hip, spine and arm fractures). Data on incident fracture during a five year observation period were available for those who had completed the follow-up. Incident fracture during the study period was coded as 0 or 1 for absence and presence of fracture and the type of fracture was ignored. Four of 8 Q-variant carriers sustained incident fracture whereas the fracture rate was 17.8% in 1036 subjects with the normal 23Q RUNX2 allele. Q-variant carriers were significantly more likely (Fisher's exact test, p = 0.036) to be in the fracture category (OR 4.7 with 95% CI 1.2 to 19.2). Overall, 6 of 8 Q-variants from the Western Australian study had prior or incident fractures. If the Geelong fracture study (n = 598) is combined with Western Australian study subjects who had ever fractured (n = 426), 1024 Australian subjects are available who had sustained fracture. Combining Western Australian [25] and GOS [39] subjects who had not reported fracture gave 1478 Australian non-fracture subjects. Despite the fact that no age matching was done, there was a significant increase (p = 0.036) in Q-variants within the fracture category in this comparison. Overall, of 22 Q-repeat variants identified, 12 had sustained some form of bone fracture, including the clinically important sites of femur, hip, spine and arm. Within the 30Q variants, three of five had fractured.

### Reduced capacity to transactivate RUNX2 target genes

Expression vectors were constructed to express 23Q, 16Q and 30Q variants of RUNX2. Significantly lower transactivation of target gene promoters occurred after transfection for 16Q and 30Q compared to the wild type 23Q was observed using a semi-synthetic RUNX2 reporter construct (pRRE) in mouse fibroblastic NIH3T3 cells (p = 0.002 and 0.016, for 16Q and 30Q, respectively, Fig. 1B). Similar data was obtained using the human osteocalcin promoter driving luciferase (pOSLUC) in HEK293 cells (Fig. 1C). Western blot of total protein from transfected NIH3T3 cells demonstrated the expected differences in apparent molecular weight in PAGE while there was no evidence a difference in relative abundance of Q-variants compared to 23Q wild type. Control cells transfected with empty vector showed no immunoreactivity (Fig. 1D).

### Comparison with CCD-related RUNX2 mutants

**Figure 2:**
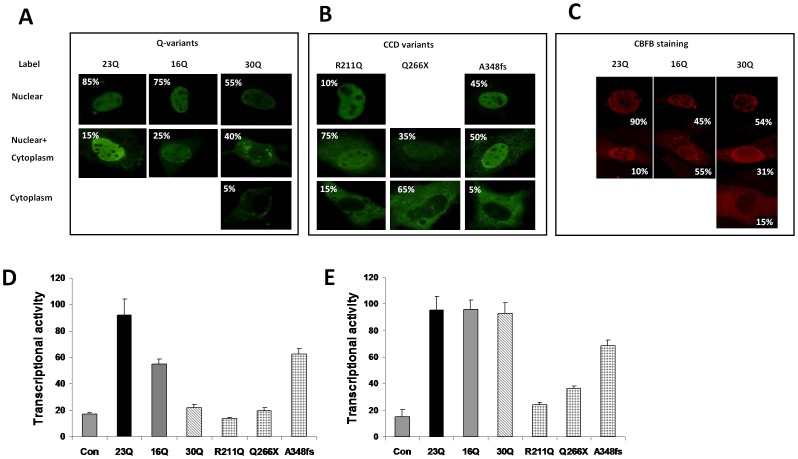
RUNX2 Q-variants have reduced nuclear localization and transactivation activity. **A**. Expression constructs were transfected into NIH3T3 cells and RUNX2 detected using confocal immunofluorescence, counts were then made of the number of cells with staining in the nuclear or cytoplasmic compartment, or both. The figures show representative cells and the insert percentage numbers indicates the proportion of cells that show that particular staining location. Panel **A** shows Q-variant forms of RUNX2 and panel **B** shows RUNX2 mutants that are associated with the condition cleidocranial dysplasia (CCD). **C**. Nuclear or cytoplasmic localization of CBFB in cells transfected with RUNX2 Q-variant constructs. **D**. RUNX2 Q-variants compared with CCD-associated RUNX2 mutants, tested against the p147 mouse osteocalcin construct. In this assay Q-variants 16Q and 30Q have a deficit of transcriptional activity that overlaps that of CCD-associated variants. **E.** Co-transfection of CBFB expression vector with RUNX2 Q-variants completely overcomes the defect in transcriptional activity detected by the p147 promoter. In contrast, the transcriptional activity of CCD-associated RUNX2 mutants on the p147 construct is not rescued by CBFB co-transfection. The transcriptional activities of 16Q and 30Q alleles were compared to wild type 23Q RUNX2 and known CCD-related mutants using an *in vitro* target gene reporter assay consisting of the proximal mouse osteocalcin promoter (p147) driving luciferase target gene construct and transfected with expression vector for RUNX2 variants in mouse NIH3T3 fibroblast-like cells. In **D** and **E**, transactivation data are not percentages relative to 23Q wild type, but arbitrary units related to the ratio of firefly luciferase to Renilla luciferase. Key to symbols: Con, control cells with target gene promoter p147 but transfected with empty expression vector; 23Q, wild type; 16Q and 30Q, Q variants; R211Q, missense mutation at residue 211; Q266X, early termination at residue 266; A348fs, frames shift mutation at alanine residue 348.

CCD is a disease where severe inactivating mutations of *RUNX2* exist. Some CCD-related RUNX2 mutants have no transactivation capacity while others, related to milder phenotypes, have a residual activity. CBFB is a heterodimer binding partner of RUNX2, known to increase DNA binding. The ability of RUNX2 variants to co-localise with endogenous CBFB in either nuclear, and/or cytoplasmic compartments was assessed by confocal immunomicroscopy with simultaneous staining with RUNX2 and CBFB with different fluorophores (Fig. 2 A–C). Cells were counted with exclusively nuclear staining, nuclear plus cytoplasmic and cytoplasmic only staining. The data were expressed as percentages of cells counted with a particular type of staining. For RUNX2, more cells with cytoplasmic staining were observed in transfected cells with 16Q and 30Q variants than expected compared with wild type 23Q (p = 0.007), with the 30Q having the stronger effect (p = 0.01). The majority (90%) of cells transfected with wild type 23Q RUNX2 also showed nuclear localization of CBFB: the other 10% represented cells with both nuclear and cytoplasmic staining. For transfected 16Q variant, 75% of positive cells showed nuclear RUNX2 while only 45% showed nuclear CBFB (p = 7.5×10^−5^) with the difference appearing in the combined nuclear and cytoplasmic compartment (Fig. 2C). For transfected 30Q, the difference in CBFB binding was not as great: 64% showed nuclear RUNX2 while 54% of cells were also being positive for nuclear CBFB (p = 0.002) with a 10% increase in the nuclear and cytoplasmic compartment for CBFB.

The transactivation functions of the Q-variants were analysed using a third RUNX2 target gene reporter assay and activity compared to RUNX2 mutants known to cause CCD. Using the mouse osteocalcin reporter (p147) in NIH3T3 mouse fibroblastic cells: 16Q and 30Q variants had reduced capacity to transactivate and this was a stronger effect than had been observed in the prior reporter-cell line combinations (Fig. 2D). The depressed transactivation ability of the 30Q variant was approximately the same as that observed in the Q266X CCD mutant, whereas the 16Q variant was similar to that seen in the A348fs mutant. Exogenous transfected CBFB enhanced wild type RUNX2 activity and rescued, to a large extent, the transactivation activity on the p147 target promoters when Q-variants were considered (Fig. 2E). Under the same conditions, the activity of CCD-related RUNX2 mutants was not rescued fully by co-transfection of CBFB partner (Fig. 2E).

Endogenous CBFB nuclear translocation was significantly related to RUNX2 Q-variants when observed using confocal microscopy (Fig. 2 A–C). Despite the fact that endogenous CBFB localization might be altered in RUNX2 variants, we reasoned that transfection of CBFB expression vector, may alter the phenotype of the Q-variants by augmenting endogenous CBFB levels. Expression vector for CBFB was transfected with Q-variants and activity on the p147 target in NIH3T3 cells examined.

### Vitamin D receptor (VDR) and Q variants

**Figure 3:**
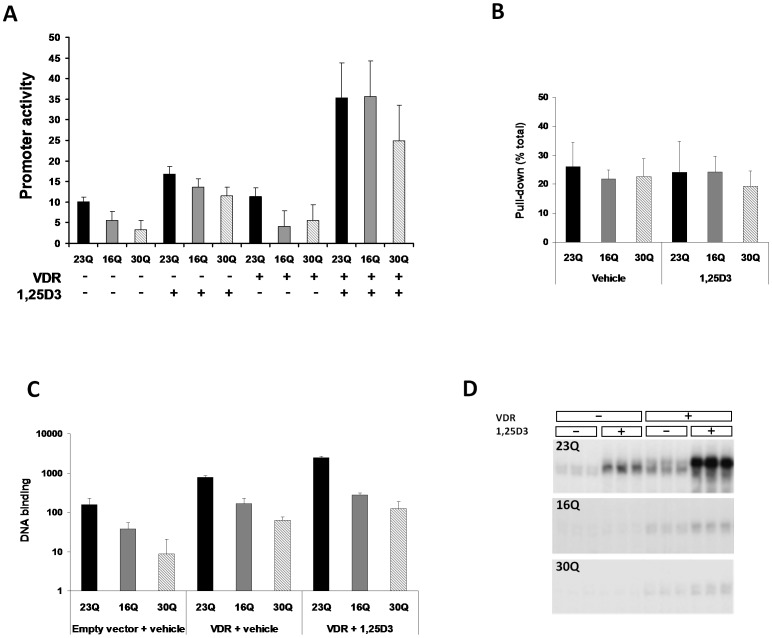
RUNX2 Q-variant interaction with the vitamin receptor (VDR). **A**. Transfected VDR, activated by 1,25(OH)_2_D_3_, can overcome the deficit in Q-variant transcriptional activity on a target human osteocalcin promoter. Although 16Q and 30Q variants show significantly reduced transactivation in NIH3T3 cells, as seen previously, transfected VDR alone does not alter the significant defect, however activation of VDR by the natural ligand 1,25(OH)_2_D_3_, results in essentially no difference in activation on the target gene promoter between the Q-variants. Promoter activity refers to human osteocalcin promoter construct driving fire fly luciferase (pOSLUC). Transactivation units are arbitrary based on the ratio of fire fly to Renilla luciferase. Empty vector controls are not shown but had activity of about one half unit on this graph. **B.** There was no detectable difference in the affinity of [^35^S] RUNX2 Q-variants for binding labelled VDR protein in *in vitro* pull-down assays where VDR was used to pull down ^35^S labelled RUNX2 protein via an affinity column. **C.** The ability of *in vitro* translated RUNX2 Q-variants to bind a RUNX2 DNA element from the mouse osteocalcin promoter (known as ORE) is enhanced by VDR in a ligand dependant manner. DNA binding was measured by phosphor analysis of labelled bands in gel shift assays after 5% PAGE. A difference in binding capacity existed on this element. Although addition of VDR or VDR and 1,25(OH)_2_D_3_, resulted in a increased total binding, in a ligand dependent manner, there was still a difference in Q-variants relative to 23Q. **D**. Images show gel shift data, with triplicates, RUNX2 variants indicated on the image and above the additional proteins added. Data suggest a difference in DNA binding and interaction with VDR. 1,25D3 in figure indicates 1,25(OH)_2_D_3_.

In order to test the interaction of VDR with RUNX2 Q-variants in cells, the human osteocalcin promoter luciferase construct (pOSLUC) was transfected into NIH3T3 fibroblasts, with or without VDR expression vector and with or without 1,25(OH)_2_D_3_ and RUNX2 expression vector (Fig. 3A). Differences in target promoter activity were once again observed, as described above, with 16Q and 30Q showing significantly lower activity compared to wildtype 23Q (Fig. 3A). As expected, transfection of VDR resulted in induction of pOSLUC. In the presence of 45 ng RUNX2 vector, 10 ng VDR vector and 1,25(OH)_2_D_3_ (at 10^−8^ M) approximately 63 fold induction of the target vector occurred compared to empty vector vehicle treated cells (Fig. 3A). In the absence of 1,25(OH)_2_D_3_, transfected VDR had no significant effect on promoter activity. Treatment of cells with 1,25(OH)_2_D_3_, in the absence of transfected VDR, resulted in some induction of pOSLUC, consistent with activation of endogenous VDR. Induction by 1,25(OH)_2_D_3_ was increased in the presence of transfected VDR (p = 1.7×10^−10^). In the absence of 1,25(OH)_2_D_3_, both 16Q and 30Q variants had significantly lower target gene promoter activity compared to 23Q wild type construct (23Q versus 16Q, p = 1.1×10^−5^; 23Q versus 30Q, p = 4.0×10^−6^), regardless of the presence or not of transfected VDR. In contrast, in the presence of 1,25(OH)_2_D_3_ and transfected VDR the difference between 23Q and other Q-variant constructs measured by osteocalcin promoter activity was eliminated or reduced (23Q versus 16Q, p = 0.95; 23Q versus 30Q, p = 0.08).

VDR is a RUNX2 partner protein that can be activated by ligand, 1,25(OH)_2_D_3_. To examine the interaction of VDR and RUNX2 variants, *in vitro* translated proteins were made and tested in glutathione-S transferase (GST) pull down reactions for differences in protein-protein interaction. Unlabelled GST-VDR fusion construct was used to pull down ^35^S labelled Q variant RUNX2 proteins (Fig. 3B). Under these conditions, GST-VDR demonstrated a strong interaction with RUNX2 (approximately 25% of label being recovered in pull down) but no significant difference in capacity to pull down RUNX2 Q-variants (p = 0.08), suggesting that protein-protein interactions of RUNX2 and VDR are unaffected by either variation in the glutamine repeat or the GST moiety added to VDR. There was no difference in the amount of GST-pull down signal in the presence or absence of 1,25(OH)_2_D_3_ ligand.

Quantitative gel-shift assays were done using *in vitro* translated RUNX2 variants and a synthetic RUNX2 element from the mouse osteocalcin gene. These data showed a significant difference in the binding capacity of the Q-variants to the response element, suggesting that variation in the Q repeat can influence DNA binding, at least on some elements. *In vitro* conditions for ligand (1,25(OH)_2_D_3_) dependent VDR binding to DNA were previously described [36]. Under these conditions, the RUNX2 response element binding activity was increased by addition of *in vitro* translated VDR protein and further enhanced by addition of 1,25(OH)_2_D_3_. Despite increased DNA affinity in the presence of VDR and 1,25(OH)_2_D_3_ a significant difference in the *in vitro* DNA binding activity of Q-variants persisted such that 16Q and 30Q variants were less active than 23Q wild type (Fig. 3 C, D).

## Discussion

Various lines of evidence suggest that the RUNX2 Q/A repeat region has functional polymorphism. We screened samples from various studies for Q-repeat mutations in RUNX2. A total of 26 Q-variant carriers were identified, involving 24 genetically unique individuals. The Q-variants did not present with cleidocranial dysplasia (CCD) and thus we hypothesized that the Q-variants may alter another skeletal phenotype such as BMD. Overall, Q-variants were significantly lower for BMD measures including bone ultrasound attenuation of the calcaneus, and DEXA measures of BMD at several sites. After pooling BMD measures from all studies, using Z scores, an overall estimate of the effect of Q-variant status was obtained. The genetic effect of Q-variants was around 0.7 Z-score (SD) reduction in bone density whether measured by DEXA or ultrasound. These data confirm that the Q-tract of RUNX2 has a reasonably high rate of population variation and furthermore, suggest that this variation manifests in the phenotype of lower BMD. Q-variant heterozygotes were detected at around 0.4%, or around 40,000 per ten million inhabitants. Q-variant homozygotes are expected to be infrequent; but such homozygotes may have a more severe phenotype with a mean effect of −1.4 Z-scores, if the genetic effect is additive. Whether Q-insertions have a more extreme phenotype than deletions, as suggested by the BMD data, remains to be determined in larger populations.

New repeat variants can occur through strand slippage during replication of repeat sequences such as that encoding the RUNX2 Q-repeat. Expansion of Q-repeats is known in other diseases such as Kennedy's disease where expanded androgen receptor repeats result in motor neuron disease [40] and, incidentally, the length of the repeat has been related to transcriptional function [41]. Long Q-repeats are thought to adopt β-sheet structures [42] that coil into a nanotube by forming a super helix with a critical point of increased stability at a length of 30Q [43]. This might suggest that the super structure of the Q-repeat, rather than the length, is important for altered function. Although the Q-variants observed in this study were either 30Q or less, it seems possible that longer repeat expansion of RUNX2 may be observed and may be associated with some other pathology. Interestingly, the most frequent variants observed are 16Q and 30Q, each being 7 amino acids difference from the standard wild type 23Q allele. This represents a deletion or expansion of 21 base pairs in the DNA helix, being exactly two turns of the helix increase or decrease, given the accepted value of 10.5 base pairs per helical turn in DNA.

The transcriptional activities of the 16Q and 30Q mutants were analysed using three different luciferase reporter gene assays. The variants exhibited transactivation function, however at significantly reduced levels compared to wild type (23Q). The effect was of greater magnitude using a truncated mouse osteocalcin promoter, p147, where 16Q and 30Q variant activity overlapped that of some RUNX2 mutations that are associated with cleidocranial dysplasia. The reduction in transactivation function is consistent with the decrease in BMD observed. In the case of a heterozygote with a normal wild-type allele, the net effect would be expected to reflect the activity of individual alleles. The significant effect of Q-variant RUNX2 on transactivation activity in transfection assays could result from effects on DNA binding, nuclear residence and interactions with partner proteins. Nuclear residence of Q-variants was decreased significantly in transfected cells while quantitative gel shift assays suggested a difference in binding to consensus DNA elements. We cannot distinguish between the nuclear import effects and direct effects on transcriptional machinery, since we did not examine *in vitro* transcription. Regardless of the mechanism, it seems plausible that the quantitative reduction in the transactivation capacity of RUNX2 is associated with overall decrease in BMD seen in human carriers. Accumulated over a lifetime, this may be enough to explain the observed effects on bone density. On the other hand, only one semi-synthetic and two natural target genes were tested, so the effect of Q-variants on other genes is not known. The vitamin D receptor (VDR) is ligand activated, is known to bind RUNX2 by interaction on the osteocalcin promoter [44], and has potent direct effects on bone cells. In the human, but not in mouse, VDR activates osteocalcin through a vitamin D response element. In this case, partial recovery of the transactivation potential of the RUNX2 Q-variants on the human osteocalcin promoter was observed on co-transfection and activation of VDR with the ligand 1,25(OH)_2_D_3_.

Are the Q-variants related to fracture? While it is suggestive that a significant effect on incident fracture was observed within the Western Australian study, this outcome should be viewed with caution due to the small numbers of Q variants. In addition, the apparent enrichment of Q-variants in post-menopausal clinic fracture cases (four variants in 303 cases genotyped) should also be considered with caution. Given the rare nature of these Q-variants, much larger studies are required to determine the relationship with fracture with precision. A frequently quoted proposition is that for every one standard deviation decrease in BMD, a person increases two fold in liability for fracture. If the effect on BMD of Q-variants is similar to the measured amount (0.7SD deficit), we can expect an increase in liability to fracture of slightly less than two-fold, if the effect of a gene such as RUNX2 on fracture acts solely via BMD. Based on the observed allele frequency, very large studies indeed (10,000 cases and controls) would be required for reasonable power test the hypothesis of association with fracture at 99% confidence for an odds ratio of around 2. If the hypothetical effect on fracture is greater than that suggested by BMD differences, then it may be possible to detect an effect in smaller numbers.

Severe RUNX2 mutations that cause CCD have a more profound effect in transfection assays, usually abolishing transactivation [6], [7], [19], [20]. Milder phenotypes are associated with RUNX2 mutants that have some transactivation function. In some transactivation assays, notably using the p147 mouse osteocalcin promoter, the 30Q RUNX2 variant resulted in transactivation in a manner similar to CCD mutant RUNX2 forms (Q266X), suggesting a phenotype similar to CCD in terms of transfection. The subjects that carry Q-repeat mutations do not present with CCD, thus the Q-variant proteins transactivate target promoters at levels that are high enough to avoid the manifestations of CCD but at levels that reduce bone density within the normal range. Alternatively, some other mechanism may keep the phenotype out of the range of CCD, such as the CBFB or VDR interactions that were investigated in this study. The transfection data are consistent with this hypothesis. Our constructs were based on the RUNX2 protein that starts MRIPV, an isoform that is expressed off the second promoter of RUNX2. At least in mouse, and possibly in human, an isoform beginning MASNS is expressed off the first promoter. We have not tested the effect of Q-variants within the context of the isoform RUNX2-II, expressed off the first promoter. In addition, recent evidence suggests that Q-repeat regions contain an activity similar to a trans-activation domain [45], [46]. Furthermore, RUNX2 Q/A repeat variation is related to skull shape in carnivores [25], [26] suggesting that functional polymorphism is associated with this repeat: unfortunately no data on facial characteristics or skull shape was taken from the subjects presented in this study. It is also notable that the RUNX2 locus shows evidence of intense evolutionary selection in the comparison between Neanderthal and modern human DNA sequences [47]. How this relates to functional change remains to be established. Our data support a role for the Q-repeat in contributing to transactivation activity, although we have no data on a biochemical mechanism. It seems reasonable to conclude that a number of activities are altered to arrive at a change in target gene transactivation: including nuclear localization, DNA binding and possibly the activity of the Q-repeat as a transactivation function. The data suggests lower transcriptional activity of RUNX2, contributing to lower bone density and possibly flow-on increased risk of fracture, through reduced transcriptional activity in osteoblasts.

There are several limitations of this study. No subjects were rejected from the collections for any reason prior to genotyping although some DNA failed to amplify for technical reasons. Recruitment bias was minimised as far as could be done in such clinical collections and there is no reason to suspect that genetic stratification might have influenced the outcome, although this was not formally tested. Similar results were observed in different locations, although all populations were essentially Caucasian. Bone phenotype data were derived from different commercial devices in each population and were compared through the use of standardized Z scores or Monte Carlo modelling; which should remove device-related differences. The study was limited to female Caucasians. The statistical outcome observed was not dependent on modelling covariates, although adjusted standardized values are presented. While statistically significant, these outcomes do not extend to the whole genome level of significance used in genome scans, although this study was not designed as a genome scan. The Q-variants observed in this study may be new mutations, although intergenerational studies are needed to verify this. If these are new mutants such a mutable locus may not be detected in a whole genome association study, as new mutations would be sporadically associated with genetic markers used in genome scans studies such as haplotype blocks. We have not formally established that these variants are new mutations, although the range of alleles (15Q, 16Q, 18Q and 30Q) suggests new mutations and these are compatible with strand slippage models of DNA replication through repeated sequences. Furthermore, the biochemical data based on transfections for Q-variants were tested only in 16Q and 30Q types and only within the context of RUNX2-I isoform. Biochemical effects of Q-variants may be different in the RUNX2-II isoform, which is more osteoblast specific [48] and has a differential interaction with CBFB [49] compared to RUNX2-I. Three different target constructs were tested, with similar results, although other target promoters may give different results. Finally, interactions of VDR and RUNX2 were tested in artificial constructs with a GST moiety, and tested on a single DNA element, so differences observed may not relate to the situation in the human and on other promoters.

In conclusion, the lines of evidence presented here suggest that variation in the glutamine repeat of RUNX2 has functional consequence. Significantly different phenotypic values for bone density parameters were observed in individuals heterozygous for the Q-variants. Significant functional differences were observed in assays of transcription factor activity on different target gene promoters. Furthermore, an alteration in both *in vitro* DNA binding activity and nuclear residence was observed, suggesting a number of different cumulative effects might occur with these variants. A partial correction of the defect in Q-variant transcriptional activity was observed in studies of interaction with two nuclear partners, CBFB and VDR, which are candidates for increasing residence of the RUNX2 variant on DNA. In addition, due to the large number of potential carriers, this genetic marker may be of some interest in the clinical setting. Intriguingly, transfection of the VDR, in the presence of active 1,25(OH)_2_D_3_ overcame some of the *in vivo* defect in RUNX2 Q-variant, suggesting a possible route for clinical studies of Q-variant carriers or at least future studies of the bone phenotype of Q-variants relative to vitamin D status.

## References

[1] McGuigan FE, Murray L, Gallagher A, Davey-Smith G, Neville CE, et al (2002) Genetic and environmental determinants of peak bone mass in young men and women. J Bone Miner Res 17: 1273–1279. 12096841 10.1359/jbmr.2002.17.7.1273

[2] Kanis JA, Melton LJ III, Christiansen J, Johnston CC, Khaltaev N (1994) The diagnosis of osteoporosis. J Bone Miner Res 9: 1137–1141. 7976495 10.1002/jbmr.5650090802

[3] Chan MY, Nguyen ND, Center JR, Eisman JA, Nguyen TV (2012) Absolute fracture-risk prediction by a combination of calcaneal quantitative ultrasound and bone mineral density. Calcif Tissue Int 90: 128–136. 22179560 10.1007/s00223-011-9556-3

[4] Ducy P, Zhang R, Geoffroy V, Ridall AL, Karsenty G (1997) Osf2/Cbfa1: a transcriptional activator of osteoblast differentiation. Cell 89: 747–754. 9182762 10.1016/s0092-8674(00)80257-3

[5] Komori T, Yagi H, Nomura S, Yamaguchi A, et al (1997) Targeted disruption of Cbfa1 results in a complete lack of bone formation owing to maturational arrest of osteoblasts. Cell 89: 755–764. 9182763 10.1016/s0092-8674(00)80258-5

[6] Otto F, Thornell AP, Crompton T, Denzel A, Gilmour KC, et al (1997) Cbfa1, a candidate gene for cleidocranial dysplasia syndrome, is essential for osteoblast differentiation and bone development. Cell 89: 765–771. 9182764 10.1016/s0092-8674(00)80259-7

[7] Mundlos S, Otto F, Mundlos C, Mulliken JB, Aylsworth AS, et al (1997) Mutations involving the transcription factor CBFA1 cause cleidocranial dysplasia. Cell 89: 773–779. 9182765 10.1016/s0092-8674(00)80260-3

[8] Enomoto H, Enomoto-Iwamoto M, Iwamoto M, Nomura S, Himeno M, et al (2000) Cbfa1 is a positive regulatory factor in chondrocyte maturation. J Biol Chem 275: 8695–8702. 10722711 10.1074/jbc.275.12.8695

[9] Vaughan T, Pasco JA, Kotowicz MA, Nicholson GC, Morrison NA (2002) Alleles of RUNX2/CBFA1 gene are associated with differences in bone mineral density and risk of fracture. J Bone Miner Res 17: 1527–1534. 12162506 10.1359/jbmr.2002.17.8.1527

[10] Vaughan T, Reid DM, Morrison NA, Ralston SH (2004) RUNX2 alleles associated with BMD in Scottish women; interaction of RUNX2 alleles with menopausal status and body mass index. Bone 34: 1029–1036. 15193550 10.1016/j.bone.2004.02.004

[11] Napierala D, Garcia-Rojas X, Sam K, Wakui K, Chen C, et al (2005) Mutations and promoter SNPs in RUNX2, a transcriptional regulator of bone formation. Mol Genet Metab 86: 257–268. 16140555 10.1016/j.ymgme.2005.07.012

[12] Doecke JD, Day CJ, Stephens AS, Carter SL, van Daal A, et al (2006) Association of functionally different RUNX2 P2 promoter alleles with BMD. J Bone Miner Res 21: 265–273. 16418782 10.1359/JBMR.051013

[13] Bustamante M, Nogués X, Agueda L, Jurado S, Wesselius A, et al (2007) Promoter 2 -1025 T/C polymorphism in the RUNX2 gene is associated with femoral neck BMD in Spanish postmenopausal women. Calcif Tissue Int 81: 327–332. 17878995 10.1007/s00223-007-9069-2

[14] Ermakov S, Malkin I, Keter M, Kobyliansky E, Livshits G (2008) Family based association study of polymorphisms in the RUNX2 locus with hand bone length and hand BMD. Ann Hum Genet 72: 510–518. 18373722 10.1111/j.1469-1809.2008.00441.x

[15] Lee HJ, Koh JM, Hwang JY, Choi KY, Lee SH, et al (2009) Association of a RUNX2 promoter polymorphism with bone mineral density in postmenopausal Korean women. Calcif Tissue Int 84: 439–445. 19424741 10.1007/s00223-009-9246-6

[16] Long F (2011) Building strong bones: molecular regulation of the osteoblast lineage. Nat Rev Mol Cell Biol 13: 27–38. 22189423 10.1038/nrm3254

[17] Online Mendelian Inheritance in Man, OMIM®. McKusick-Nathans Institute of Genetic Medicine, Johns Hopkins University (Baltimore, MD) (2012) World Wide Web URL: http://omim.org/#119600, CLEIDOCRANIAL DYSPLASIA; **CCD**

[18] Cohen MM Jr (2009) Perspectives on *RUNX* genes: an update. Am J Med Genet 149: 2629–2646. 10.1002/ajmg.a.33021 19830829

[19] Yoshida T, Kanegane H, Osato M, Yanagida M, et al (2002) Functional analysis of RUNX2 mutations in Japanese patients with cleidocranial dysplasia demonstrates novel genotype-phenotype correlations. Am J Hum Genet 71: 724–738. 12196916 10.1086/342717 PMC378531

[20] Zhang YW, Yasui N, Kakazu N, Abe T, Takada K, et al (2000) PEBP2alphaA/CBFA1 mutations in Japanese cleidocranial dysplasia patients. Gene 244: 21–28. 10689183 10.1016/s0378-1119(99)00558-2

[21] McMurray CT (2010) Mechanisms of trinucleotide repeat instability during human development. Nat Rev Genet 11: 786–799. 20953213 10.1038/nrg2828 PMC3175376

[22] Pineda B, Hermenegildo C, Laporta P, Tarín JJ, Cano A, et al (2010) Common polymorphisms rather than rare genetic variants of the Runx2 gene are associated with femoral neck BMD in Spanish women. J Bone Miner Metab 28: 696–705. 20407796 10.1007/s00774-010-0183-2

[23] Fondon JW 3rd, Garner HR (2004) Molecular origins of rapid and continuous morphological evolution. Proc Natl Acad Sci USA 101: 18058–18063. 15596718 10.1073/pnas.0408118101 PMC539791

[24] Sears KE, Goswami A, Flynn JJ, Niswander LA (2007) The correlated evolution of Runx2 tandem repeats, transcriptional activity, and facial length in carnivora. Evol Dev 9: 555–565. 17976052 10.1111/j.1525-142X.2007.00196.x

[25] Prince RL, Devine A, Dhaliwal SS, Dick IM (2006) Effects of calcium supplementation on clinical fracture and bone structure: results of a 5-year, double-blind, placebo-controlled trial in elderly women. Arch Intern Med 166: 869–875. 16636212 10.1001/archinte.166.8.869

[26] Henry MJ, Pasco JA, Nicholson GC, Seeman E, Kotowicz MA (2000) Prevalence of osteoporosis in Australian women: Geelong Osteoporosis Study. J Clin Densitom 3: 261–268. 11090233 10.1385/jcd:3:3:261

[27] Sanders KM, Seeman E, Ugoni AM, Pasco JA, Martin TJ, et al (1999) The age- and gender-specific rate of fractures in Australia: a population based study. Osteoporosis Int 10: 240–247. 10.1007/s001980050222 10525717

[28] Makovey J, Nguyen TV, Naganathan V, Wark JD, Sambrook PN (2007) Genetic effects on bone loss in peri- and postmenopausal women: a longitudinal twin study. J Bone Miner Res 22: 1773–1780. 17620052 10.1359/jbmr.070708

[29] Ding C, Parameswaran V, Cicuttini F, Burgess J, Zhai G, et al (2008) Association between leptin, body composition, sex and knee cartilage morphology in older adults: the Tasmanian older adult cohort (TASOAC) study. Ann Rheum Dis 67: 1256–1261. 18174218 10.1136/ard.2007.082651

[30] MacDonald HM, McGuigan FA, New SA, Campbell MK, Golden MHN, et al (2001) COL1A1 Sp1 polymorphism predicts perimenopausal and early postmenopausal spinal bone loss. J Bone Miner Res 16: 1634–1641. 11547832 10.1359/jbmr.2001.16.9.1634

[31] Tilyard MW, Spears GF, Thomson J, Dovey S (1992) Treatment of postmenopausal osteoporosis with calcitriol or calcium. N Engl J Med 326: 357–362. 1729617 10.1056/NEJM199202063260601

[32] Morrison NA, George PM, Vaughan T, Tilyard MW, Frampton CM, et al (2005) Vitamin D receptor genotypes influence the success of calcitriol therapy for recurrent vertebral fracture in osteoporosis. Pharmacogenet Genomics 15: 127–135. 15861036 10.1097/01213011-200502000-00008

[33] Zhang YW, Yasui N, Ito K, Huang G, Fujii M, et al (2000) A RUNX2/PEBP2alpha A/CBFA1 mutation displaying impaired transactivation and Smad interaction in cleidocranial dysplasia. Proc Natl Acad Sci USA 97: 10549–10554. 10962029 10.1073/pnas.180309597 PMC27062

[34] Mizushima S, Nagata S (1990) pEF-BOS, a powerful mammalian expression vector. Nucleic Acids Res 18: 5322. 1698283 10.1093/nar/18.17.5322 PMC332193

[35] Yoshida T, Kanegane H, Osato M, Yanagida M, Miyawaki T, et al (2002) Functional analysis of RUNX2 mutations in Japanese patients with cleidocranial dysplasia demonstrates novel genotype-phenotype correlations. Am J Hum Genet 71: 724–738. 12196916 10.1086/342717 PMC378531

[36] Dyer BW, Ferrer FA, Klinedinst DK, Rodriguez R (2000) A noncommercial dual luciferase enzyme assay system for reporter gene analysis. Anal Biochem 282: 158–161. 10860516 10.1006/abio.2000.4605

[37] Polly P, Herdick M, Moehren U, Baniahmad A, Heinzel T, et al (2000) VDR-Alien: a novel, DNA-selective vitamin D(3) receptor-corepressor partnership. FASEB J 14: 1455–1463. 10877839 10.1096/fj.14.10.1455

[38] Hood GM (2010) PopTools version 3.2.3 (CSIRO Canberra, Australia). Available on the internet. URL http://www.poptools.org

[39] Pasco JA, Seeman E, Henry MJ, Merriman EN, Nicholson GC, et al (2006) The population burden of fractures originates in women with osteopenia, not osteoporosis. Osteoporos Int 17: 1404–1409. 16699736 10.1007/s00198-006-0135-9

[40] Finsterer J (2010) Perspectives of Kennedy's disease. J Neurol Sci 298: 1–10. 20846673 10.1016/j.jns.2010.08.025

[41] Chamberlain NL, Driver ED, Miesfeld RL (1994) The length and location of CAG trinucleotide repeats in the androgen receptor N-terminal domain affect transactivation function. Nucleic Acids Res 22: 3181–3186. 8065934 10.1093/nar/22.15.3181 PMC310294

[42] Perutz MF, Finch JT, Berriman J, Lesk A (2002) Amyloid fibers are water-filled nanotubes. Proc Natl Acad Sci USA 99: 5591–5595. 11960014 10.1073/pnas.042681399 PMC122814

[43] Ogawa H, Nakano M, Watanabe H, Starikov EB, Rothstein SM, et al (2008) Molecular dynamics simulation study on the structural stabilities of polyglutamine peptides. Comput Biol Chem 32: 102–110. 18243803 10.1016/j.compbiolchem.2007.11.001

[44] Paredes R, Arriagada G, Cruzat F, Villagra A, Olate J, et al (2004) Bone-specific transcription factor Runx2 interacts with the 1alpha,25-dihydroxyvitamin D_3_ receptor to up-regulate rat osteocalcin gene expression in osteoblastic cells. Mol Cell Biol 24: 8847–8861. 15456860 10.1128/MCB.24.20.8847-8861.2004 PMC517904

[45] Atanesyan L, Nther V, Dichtl B, Georgiev O, Schaffner W (2012) Polyglutamine tracts as modulators of transcriptional activation from yeast to mammals. Biol Chem 393: 63–70. 22628299 10.1515/BC-2011-252

[46] Whan V, Hobbs M, McWilliam S, Lynn DJ, Lutzow YS, et al (2010) Bovine proteins containing poly-glutamine repeats are often polymorphic and enriched for components of transcriptional regulatory complexes. BMC Genomics 11: 654–665. 21092319 10.1186/1471-2164-11-654 PMC3014979

[47] Green RE, Krause J, Briggs AW, Maricic T, Stenzel U, et al (2010) A draft sequence of the Neandertal genome. Science 328: 710–722. 20448178 10.1126/science.1188021 PMC5100745

[48] Kanatani N, Fujita T, Fukuyama R, Liu W, Yoshida CA, et al (2006) Cbf beta regulates Runx2 function isoform-dependently in postnatal bone development. Dev Biol 296: 48–61. 16797526 10.1016/j.ydbio.2006.03.039

[49] Xiao Z, Awad HA, Liu S, Mahlios J, Zhang S, et al (2005) Selective Runx2-II deficiency leads to low-turnover osteopenia in adult mice. Dev Biol 283: 345–356. 15936013 10.1016/j.ydbio.2005.04.028 PMC1360182

